# Transcript profiling of *Populus tomentosa* genes in normal, tension, and opposite wood by RNA-seq

**DOI:** 10.1186/s12864-015-1390-y

**Published:** 2015-03-10

**Authors:** Jinhui Chen, Beibei Chen, Deqiang Zhang

**Affiliations:** National Engineering Laboratory for Tree Breeding, College of Biological Sciences and Technology, Beijing Forestry University, No. 35, Qinghua East Road, Beijing, 100083 P. R. China; Key Laboratory of Genetics and Breeding in Forest Trees and Ornamental Plants, Ministry of Education, College of Biological Sciences and Technology, Beijing Forestry University, No. 35, Qinghua East Road, Beijing, 100083 P. R. China

**Keywords:** Cellulose biosynthesis, Lignin biosynthesis, *Populus*, RNA-seq, Tension wood

## Abstract

**Background:**

Wood formation affects the chemical and physical properties of wood, and thus affects its utility as a building material or a feedstock for biofuels, pulp and paper. To obtain genome-wide insights on the transcriptome changes and regulatory networks in wood formation, we used high-throughput RNA sequencing to characterize cDNA libraries of mature xylem from tension wood (TW), opposite wood (OW), and normal wood (NW), in the industrial tree species *Populus tomentosa*.

**Results:**

Our sequencing generated 140,978,316 (TW), 128,972,228 (OW), and 117,672,362 (NW) reads, corresponding to 10,127 (TW), 10,129 (OW), and 10,129 (NW) unique genes. Of these, 361 genes were differentially transcribed between TW and OW (log_2_FC ≥ 1 or ≤ -1, FDR < 0.05), 2,658 differed between OW and NW, and 2,417 differed between TW and NW. This indicates that NW differs significantly from the wood in branches; GO term analysis also indicated that OW experienced more transcriptome remodeling. The differentially expressed genes included 97 encoding transcription factors (TFs), 40 involved in hormone signal transduction, 33 in lignin biosynthesis, 21 in flavonoid biosynthesis, and 43 in cell wall metabolism, including *cellulose synthase*, *sucrose synthase*, and *COBRA*. More than half of the differentially expressed TF showed more than 4-fold lower transcript levels in NW compared with TW or OW, indicating that TF abundances differed dramatically in different wood types and may have important roles in the formation of reaction wood. In addition, transcripts of most of the genes involved in lignin biosynthesis were more abundant in OW compared with TW, consistent with the higher lignin content of OW. We constructed two transcriptomic networks for the regulation of lignin and cellulose biosynthesis, including TFs, based on the co-expression patterns of different genes. Lastly, we used reverse transcription quantitative PCR to validate the differentially expressed genes identified.

**Conclusions:**

Here, we identified the global patterns and differences in gene expression among TW, OW, and NW, and constructed two transcriptomic regulatory networks involved in TW formation in *P. tomentosa*. We also identified candidate genes for molecular breeding of wood quality, and provided a starting point to decipher the molecular mechanisms of wood formation in *Populus*.

**Electronic supplementary material:**

The online version of this article (doi:10.1186/s12864-015-1390-y) contains supplementary material, which is available to authorized users.

## Background

Trees have important functions in ecosystems, and in providing feedstocks for global industry. For example, wood has uses in construction, pulp and paper, and a major potential role in biofuels as a renewable, cost-effective alternative to fossil fuels. The complex chemical makeup of wood also makes it an ideal raw material for a potential future “ligno-chemical” industry that could replace the petrochemical industry, providing raw materials for the manufacture of plastic, chemicals, food, and textiles [1]. However, our understanding of how wood develops remains far from complete, and very little is known about the cellular, molecular, and developmental processes that underlie wood formation [1].

Wood formation requires the coordinated expression of numerous structural genes involved in cell division, cell differentiation, programmed cell death, and heartwood formation and wood develops under the control of regulatory genes that remain virtually unknown [1]. Reaction wood, which develops under stress caused by branch bending or gravity, has been used as a model system for the functional genomics of wood formation, particularly in exploration of carbon partitioning between lignin and cellulose in woody plants. Tension wood (TW), which develops under tension stress, has more cellulose and less lignin and hemicelluloses than normal wood (NW); by contrast, opposite wood (OW, also termed compression wood, CW, in gymnosperm species), which develops under compression stress, has more lignin [2]. Thus, many studies have used microarray analysis of TW and OW in artificially or naturally bent trunks of *Populus* to identify key genes that contribute to the formation of reaction wood, including genes encoding plant hormones, lignin and cellulose biosynthetic enzymes, and transcription factors (TFs) and other potential regulators [3-6]. For example, a cDNA microarray analysis of TW induced by bending force in young *Populus tremula* (L.) demonstrated that TW formation involved reprogramming of carbohydrate metabolism, including increases of *PttCesA8-2* and *PttCesA3-2* transcripts and a decrease of transcripts involved in lignin biosynthesis [5]. For lignin biosynthesis, *4-Coumarate-CoA Ligase* (*4CL*) expression increases during CW formation in pine species [7] and transgenic suppression of *4CL* in *Pinus radiata* decreased lignin contents [8]. Plant hormones such as auxin, ethylene, and gibberellin also have important roles in the formation of TW [2]. For example, transgenic approaches showed that the endogenous ethylene produced in leaning trees acts as a key regulator of the asymmetrical cambial growth in *Populus* TW [9]. Recent work also reported that the formation of TW and stem gravitropism in *Acacia mangium* seedlings requires gibberellins [10].

The complex process of wood formation requires various genes and pathways; therefore, genome-wide transcriptome analysis, especially by high-throughput RNA sequencing (RNA-seq), provides a useful approach [3-6] to explore the mechanisms underlying wood formation. RNA-seq can detect rare transcripts, splice variants, and novel transcripts [11]. Moreover, RNA-seq data provide absolute transcript levels, rather than relative measurements, thus overcoming many limitations of microarray analysis [12]. To date, most studies have focused on the difference between TW and NW in artificially bent *Populus* trunks, and have used cDNA microarrays. However, little is known about transcription and regulation in *Populus* branches (TW and OW) under gravity stress, especially combined with analysis of NW using RNA-Seq.

To provide accurate and comprehensive genome-wide insights into the molecular mechanisms involved in the formation of TW, we used RNA-seq to reveal transcriptome changes in TW, OW, and NW in *Populus tomentosa* (Carr.), an important industrial species for pulp and paper in China. Our results improve our understanding of the formation of reaction wood in response to gravity, including identifying co-expression networks and TFs likely involved in the regulatory network controlling cellulose and lignin biosynthesis. To the best of our knowledge, this study is the first to characterize the xylem transcriptome of *P. tomentosa* using RNA-seq, and may serve as a foundation for further studies of wood formation, particularly the formation of special wood in *Populus*.

## Methods

### RNA isolation, library construction, and Solexa sequencing

As biological replicates, this study used three individuals, 30-year-old clones from one genotype of *P. tomentosa*. The three trees were planted in the national nursery of Guan Xian County (Shandong Province, China; N36°28′28″, E115°26′17″) and were not treated to induce any stress conditions. Mature xylem tissue samples from NW, TW, and OW (normal, tension, and opposite wood) were isolated from the same individual to enable comparisons in the same genetic background. From each plant, we chose three branches that are in the same side of the tree and similar diameter (about 8 cm), and were more than 10 years old. TW (upper side of the branch) and OW (lower side of the branch) were collected from the same branch, using a sharp chisel and after removing the bark of the sampling area, following the method described in Li *et al*. [13]. NW, which represents the stem xylem, was isolated from the same side of the tree at breast height, approximately one meter above the ground. All the samples were about 2 cm × 1 cm and 4–5 mm deep. Samples were collected in the morning, immediately frozen in liquid nitrogen, and stored at −80°C for isolation of RNA. Total RNA was isolated by a modified CTAB method [14] with isopropanol instead of lithium chloride for RNA precipitation. DNase was applied to eliminate any genomic DNA in the total RNA. RNA quality was monitored using a NanoDrop ND-1000 and Agilent Bioanalyzer 2100. Extracted RNA was used for RNA library construction. First, the high-quality total RNA was purified with the RNeasy micro kit (Cat#74004, Qiagen). Purified total RNA was processed with the TruSeq RNA Sample Preparation Guide to build the cDNA library. The sequencing of cDNA was carried by Shanghai Biotechnology Corporation (Shanghai, China) using Illumina HiSeq 2000 following the cBot User Guide and HiSeq 2000 User Guide with the paired-end program.

### Transcriptome mapping and differentially expressed transcripts

Pre-processing and assembly of the raw sequence data were conducted using fastx (version 0.0.13), and included the removal of low-quality sequence fragments caused by the fluorescence instrument, reads with low overall quality, the 3′ end base 10 below the quality score of Q = 10 (Q = -10log^error_ratio^), reads containing N blur, any adapter sequences, and any sequences shorter than 20 nucleotides. TopHat (version:2.0.4) [15] was used to map the clean reads to the *Populus trichocarpa* genome by spliced mapping, allowing 2 bases of mispairing and multiple hits ≤10, according to Ensembl plant15 JGI2.0 (ftp://ftp.ensemblgenomes.org/pub/plants/release-15/fasta/populus_trichocarpa/dna/Populus_trichocarpa.JGI2.0.15.dna.toplevel.fa.gz). Cufflinks (version 2.0.2) [16] was used to calculate the expression of transcripts. The FPKM (Fragments Per Kilobase of exon model per Million mapped reads) was defined as follow:

$$ \mathrm{FPKM}=\frac{\mathrm{transcript}\ \mathrm{reads}}{\mathrm{transcript}\ \mathrm{length}\times \mathrm{total}\ \mathrm{mapped}\ \mathrm{reads}\ \mathrm{in}\ \mathrm{run}}\times {10}^9 $$

The fold change (FC) for NW *vs* TW, for example, equals the FPKM of NW divided by FPKM of TW, and so on. The differentially expressed genes were selected using log_2_FC ≥ 1 or FC ≤ -1 and FDR < 0.05 (false discovery rate control, q-value).

### Gene annotation and construction of the co-expression network

Gene annotations were carried out using PopGenie (http://www.popgenie.org/) [17] and gene ontology terms were analyzed using agriGO (http://bioinfo.cau.edu.cn/agriGO/index.php) [18]. The enriched GO categories were checked using an FDR-adjusted value of ≤0.05 as the cutoff for significant GO categories. The co-expression network analysis was performed in R using the weighted gene co-expression network analysis (WGCNA) package, as previously described [19]. Briefly, only differentially expressed genes involved in cellulose and lignin biosynthesis, and TFs were used to build an unsupervised co-expression based similarity matrix using Pearson’s correlation coefficient. Then the R package WGCNA version 1.35 was used to create the networks [19], which were modeled with Cytoscape 3.2 [20].

### Quantitative real time PCR (qRT-PCR)

qRT-PCR was performed as described [21], using the TaKaRa ExTaq R PCR Kit, SYBR green dye (TaKaRa, Dalian, China) and a DNA Engine Opticon 2 machine (MJ Research, Waltham, MA). Fifteen genes including cellulose and lignin biosynthesis genes (*Pt-CESA2.1*, *Pt-ATH.2*, *Pt-GLAC90.1*, *Pt-PRX1.8*, *Pt-PAL1.2* and *Pt-PAL1.3*) were validated, and the primers are shown in Additional file 1. The efficiency of the primers was calculated by performing real-time PCR on several dilutions of first-strand cDNAs. Efficiencies of the different primer sets were similar. The specificity of each primer set was checked by sequencing PCR products. The reactions were carried out in a 20 μl volume containing 2 μl of diluted cDNA, 200 nM of each primer, and PCR Master Mix with the following conditions: 95°C for 30 s, and 45 cycles of 95°C for 5 s, 58°C for 15 s, and 72°C for 20 s. Then, a thermal denaturing cycle of 95°C for 15 s and 60°C for 1 min was applied to determine the dissociation curves, which were used to verify the specificity of PCR amplifications. All reactions were run in triplicate for each sample. Relative expression levels of candidate genes were calculated by the 2^−ΔCt^ method. The results obtained for the different tissues were analyzed and standardized to the mRNA levels of poplar *ACTINII-like* (Accession number: EF145577), which shows stable expression.

## Results

### Global transcriptome analysis of the RNA-seq data

**Figure 1:**
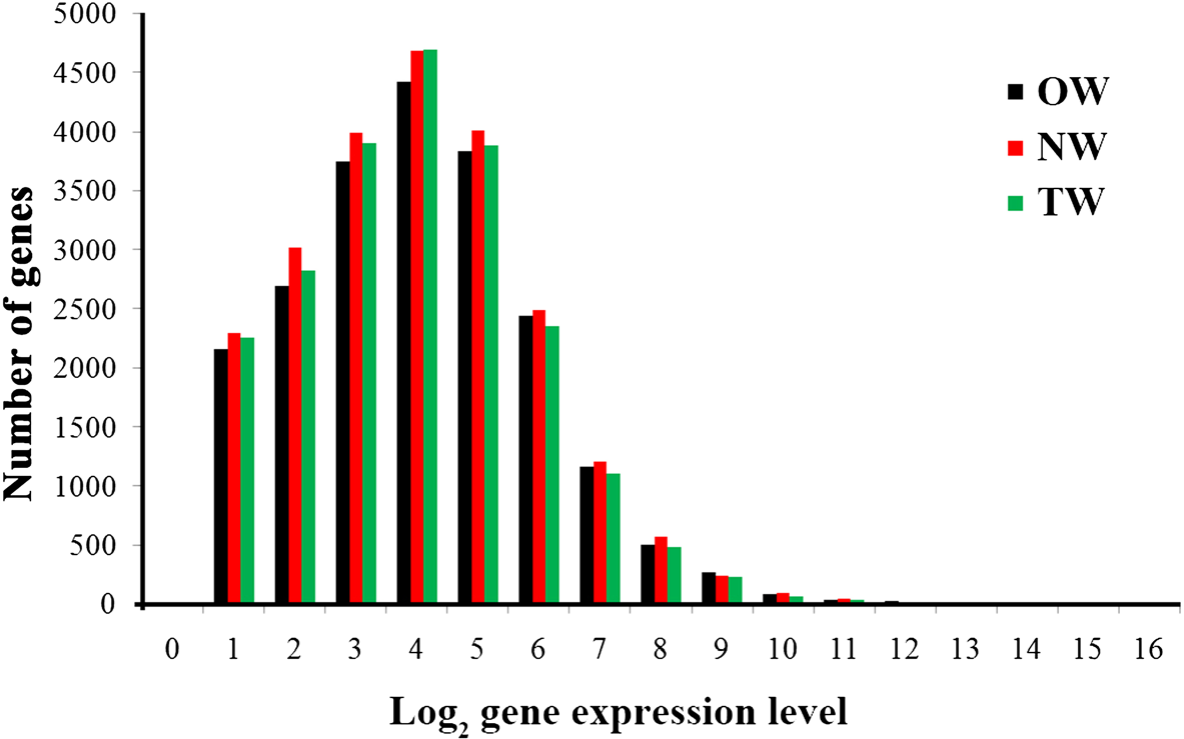
**The distribution of gene expression levels.** Total reads from each of three libraries that match to the *Populus* gene models were plotted as integrated log_2_ values. The distribution is based on the number of genes in each log_2_ gene expression category (>0 and < =16). It revealed that most of the mRNAs are expressed at low levels, with a small proportion of mRNA that is highly expressed. Also, all three libraries have similar expression patterns.

To evaluate whether the RNA-seq data are sufficient for further analysis, we first assessed their global quality. The RNA-seq generated 140,978,316 (TW), 128,972,228 (OW), and 117,672,362 (NW) reads, with 119,716,602 (TW), 108,187,750 (OW), and 101,399,718 (NW) cleaned reads remaining after trimming (Table 1). Among the total cleaned reads, 69,701,332 (TW), 64,245,293 (OW), and 59,595,595 (NW) were mapped to the *P. trichocarpa* genome with mapping ratios of 58.22% (TW), 59.38% (OW), and 58.77% (NW) (Table 2). Transcripts of length 500–1,000 bp accounted for 72.19% (TW), 70.13% (OW), and 73.58% (NW) of the reads, with averages of 690 (TW), 703 (OW), and 686 (NW), showing that the majority of transcripts are about 500–1,000 bp (Additional file 2). Based on previous studies [22,23], these results indicated that our RNA-seq results were sufficient to detect most expressed genes and transcripts for subsequent quantitative analysis. Finally, 10,127 (TW), 10,129 (OW) and 10,129 (NW) genes were identified (Additional file 3). Examination of the transcript levels (by RPKM) showed that most of the mRNAs occurred at low levels, with only a small proportion of highly expressed mRNAs. For example, most genes (4,678) distributed around 3 (>3 and ≤ 4, log_2_ gene expression) and 0.69% of the genes (157 of 22,638) showed high expression of more than 10 in NW (Figure 1). All genes also showed similar patterns of expression in the three libraries (Figure 1). These analyses indicate that the RNA-seq experiments conducted in this study were sufficiently reliable for the identification of genes that are differentially transcribed in the three wood tissues.

**Table 1:** **Summary of RNA-seq for NW, TW, and OW**

**ID**	**Number of raw reads**	**Number after intensity trimming**	**Number after quality trimming**	**Number after adaptor trimming**	**Clean reads**	**Clean ratio**
OW	128,972,228	119,920,406	115,056,016	113,476,507	108,187,750	83.88%
NW	117,672,362	110,144,876	106,779,089	105,309,930	101,399,718	86.17%
TW	140,978,316	130,868,498	126,466,946	124,744,435	119,716,602	84.92%

**Table 2:** **Mapping result statistics in NW, TW, and OW**

**ID**	**All reads**	**Mapped reads**	**Mapped pair reads**	**Mapped broken-pair reads**	**Mapped unique reads**	**Mapped multi reads**	**Mapping ratio**
OW	108,187,750	64,245,293	45,877,722	18,367,571	60,659,907	3,586,922	59.38%
NW	101,399,718	59,595,595	42,988,244	16,607,351	56,357,511	3,239,732	58.77%
TW	119,716,602	69,701,332	49,398,004	20,303,328	65,795,616	3,907,023	58.22%

**Figure 2:**
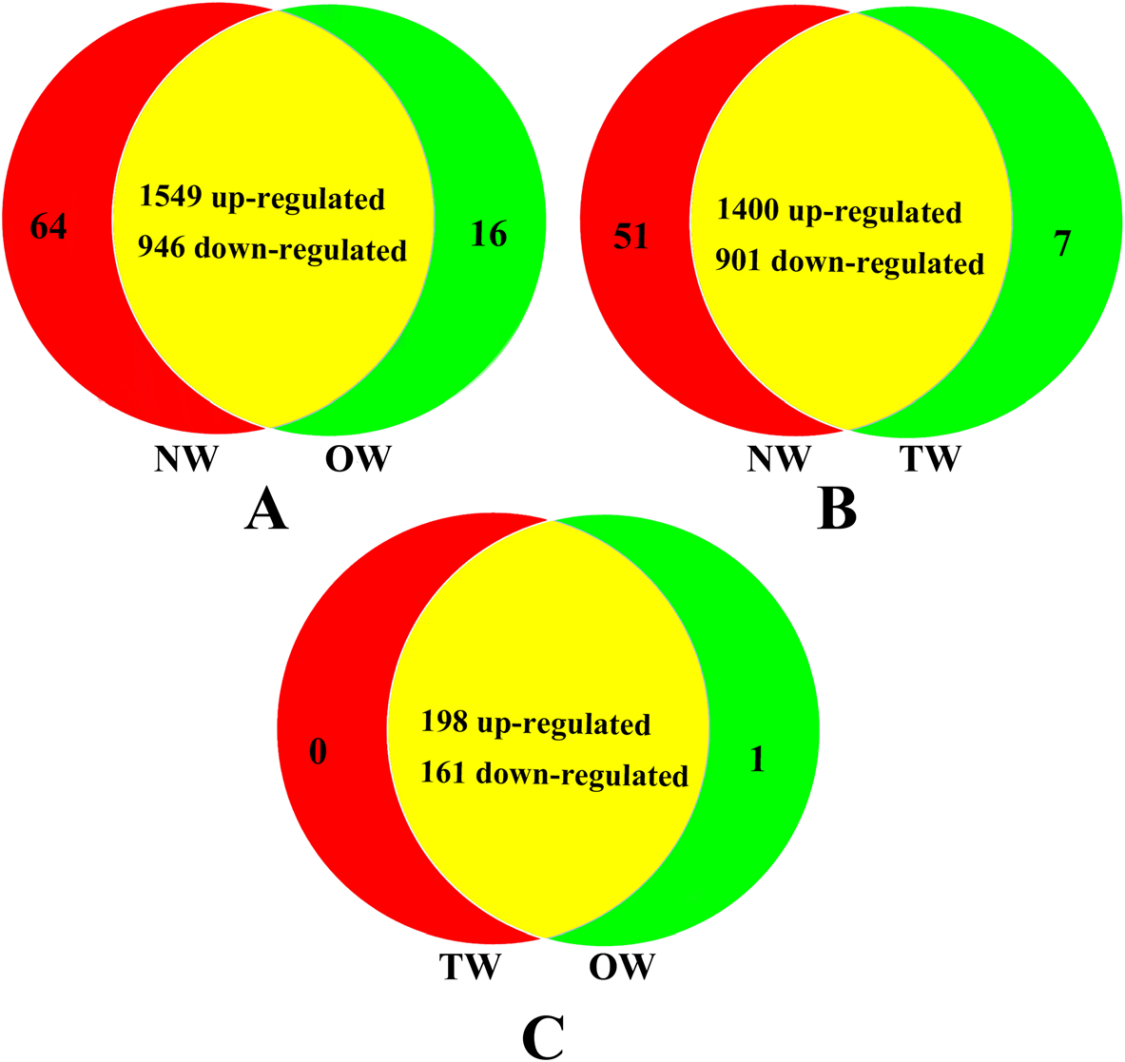
**Summary of differentially expressed genes in NW, TW, and OW.** The differentially expressed genes were selected using log_2_(FC) ≥ or ≤ -1 and FDR ≤ 0.05 including those only expressed in one library, and FDR < 0.05. **A**: The differentially expressed genes in NW and OW. **B**: The differentially expressed genes in NW and TW. **C**: The differentially expressed genes in TW *vs* OW.

To measure the changes in gene expression and find the key genes, we further selected the significantly differentially expressed genes using log_2_FC ≥ 1 or ≤ -1 and FDR ≤ 0.05; this includes genes uniquely transcribed in one library, with FDR ≤ 0.05. In total, 2,658 (NW *vs* TW), 2,417 (NW *vs* OW), and 361 (TW *vs* OW) genes were either up- or down-regulated (Additional file 4, Figure 2), reaching a total of 3,058 genes. Of the 2,658 genes that differed in NW *vs* OW, 1,613 were up-regulated, 965 were down-regulated, and 64 and 16 were uniquely expressed in NW and OW, respectively (Figure 2A). Of the 2,417 genes that differed in NW *vs* TW, 1,451 were up-regulated, 908 were down-regulated, and 51 and 7 were uniquely expressed in NW and TW, respectively (Figure 2B). Finally, of the 361 genes that differed in TW *vs* OW, 198 were up-regulated, 162 were down-regulated, and one gene was specifically expressed in OW (Figure 2C). Consequently, this analysis identified 3,058 genes that were significantly differentially expressed in the TW, OW, and NW xylem tissues.

### Functional annotation of differentially expressed genes

**Figure 3:**
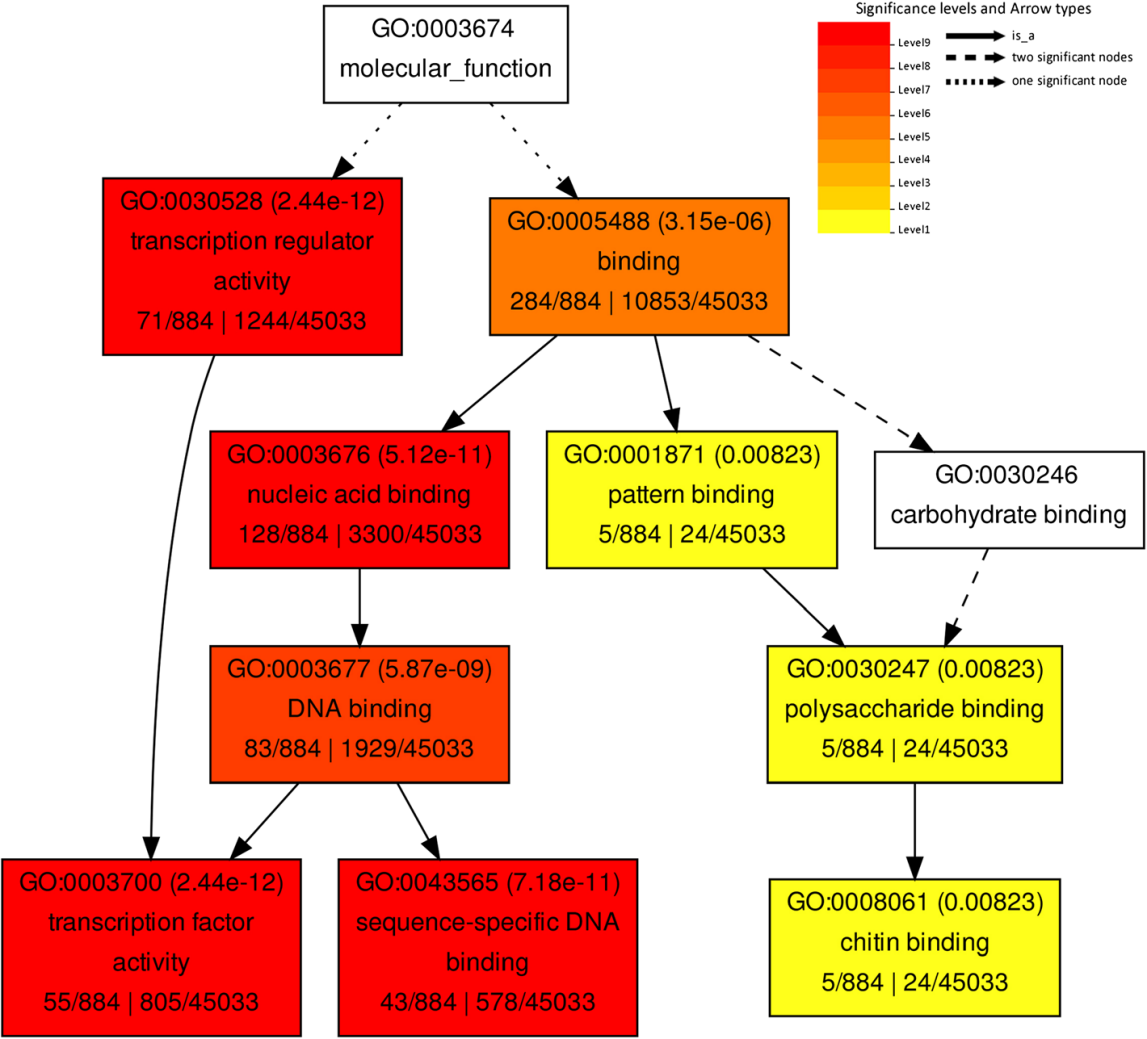
**Significant molecular functional terms for the down-regulated genes between NW and TW.** The GO terms were analyzed by agriGO using an FDR-adjusted value of ≤0.05 as the cutoff for significant GO categories.

To reveal the functions of the significant differentially expressed genes, we used GO classification to annotate them. In total, 150 significant GO terms were detected from the differentially expressed genes in NW *vs* TW in our study (Additional file 5). For “biological process” terms, terms related to “metabolic process”, “biosynthetic process”, and “assembly” were categorized. Within “molecular function”, “cytoskeletal protein binding”, and “actin binding”, as well as “S-methyltransferase activity” were identified. Notably, the “transcription regulator activity” and “transcription factor activity” terms were found in “molecular function” (Figure 3). In addition, the “nucleus” term was identified in “cellular component” for genes down-regulated in NW compared with TW.

**Figure 4:**
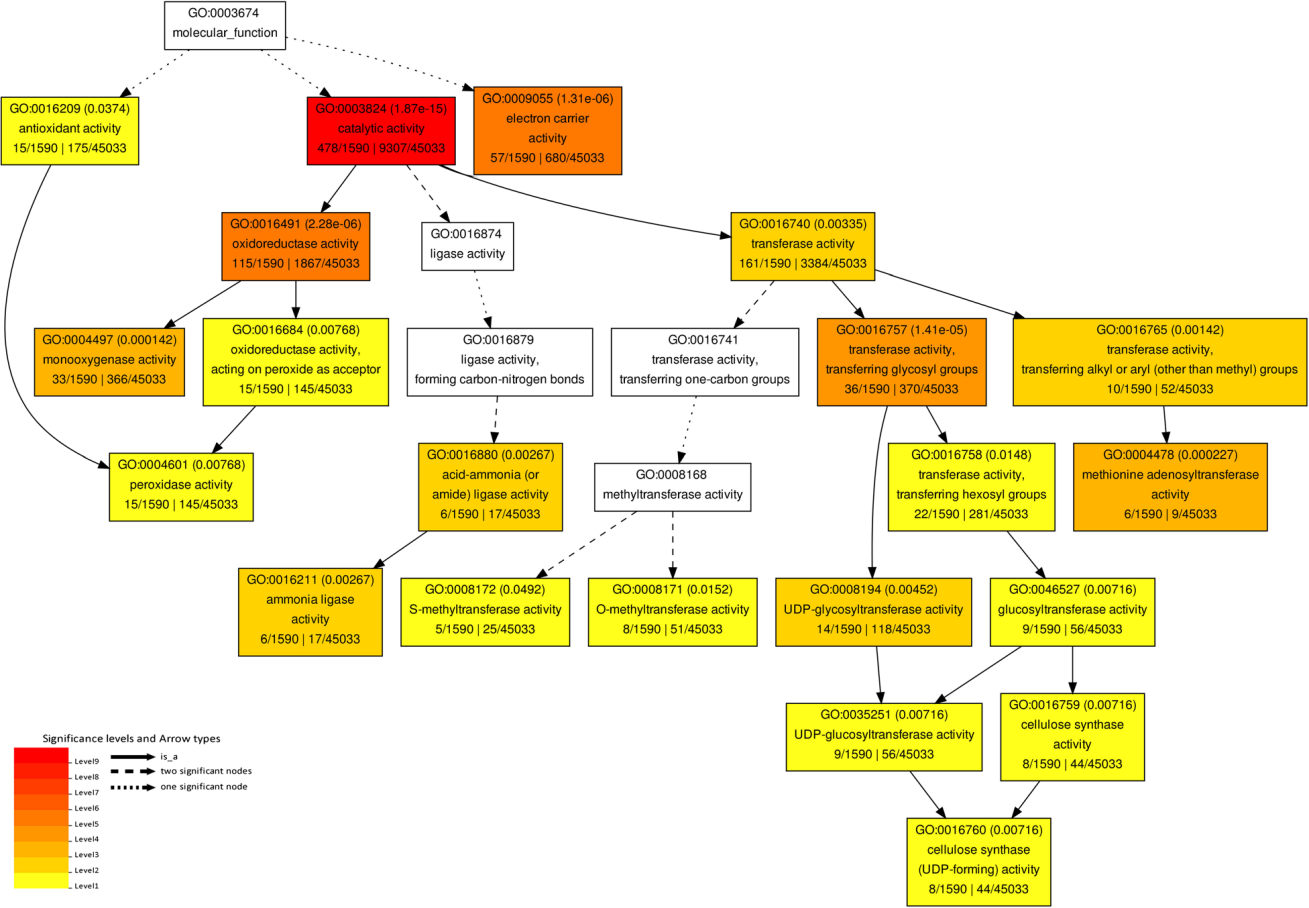
**A subset of the significant molecular functional terms for the up-regulated genes between NW and OW.** The GO terms were analyzed by agriGO using an FDR-adjusted value of ≤0.05 as the cutoff for significant GO categories.

We next characterized the differentially expressed genes in NW *vs* OW, and identified 171 significant terms (Additional file 5). Among the up-regulated genes in NW *vs* OW, 15 terms such as “response to oxidative stress” were detected. Notably, the “cellulose metabolic process” and “cellulose biosynthetic process” were both identified among the up-regulated genes in NW in comparison to TW or OW. Also, “cellulose synthase (UDP-forming) activity”, “cellulose synthase activity”, “oxidoreductase activity”, and “peroxidase activity” were only found among up-regulated genes in NW *vs* OW (Figure 4). Most of the GO terms found among the down-regulated genes in NW *vs* OW were also found in NW *vs* TW.

Notably, unlike the differentially expressed genes in NW *vs* OW, and NW *vs* TW, less information about GO terms was found for TW *vs* OW (Additional file 5). No significant terms were found in the up-regulated genes of TW *vs* OW. Only eight terms for “molecular function”, such as “cofactor binding” and “heme binding and tetrapyrrole binding”, were found in the down-regulated genes in TW *vs* OW (Additional file 5).

### Differential expression of genes involved in stress responses and transcriptional regulation

In the “response to chemical stimulus”, “response to desiccation”, “response to abiotic stimulus”, and “response to oxidative stress” terms, 34 genes were up-regulated in NW compared with TW or OW (Additional file 6). Of these, only 15 genes were annotated, including 6 genes that encode peroxidases (POs). Importantly, our study identified a large proportion of differentially expressed genes (97) related to transcriptional regulation, based on the GO terms “transcription regulator activity”, “transcription factor activity”, and “transcription repressor activity” (Additional file 6). In particular, all the putative transcriptional regulators were down-regulated in NW compared with TW and OW and included genes encoding TFs of the families: MYB, NAC, heat shock TF (HSF), AP2 domain-containing TF family protein, bZIP TF, basic helix-loop-helix (bHLH) family protein, and WRKY TF (Additional file 6). These genes also showed strong changes in expression level; of these genes, 39 and 50 genes were down-regulated more than 4-fold in NW compared with TW or OW, respectively, and 6 genes were down-regulated more than 3-fold in TW compared with OW (Additional file 6). Of these 97 TFs, six and ten TF genes involved in lignin and cellulose biosynthesis were differentially expressed in TW, OW, and NW, including *LIM*, *MYB*, *VNI*, *ASL*, and *KNAT* family members. Although both *Pt-LIM1* (NW > OW > TW) and *Pt-LIM2* (NW > TW > OW) were up-regulated in NW, *Pt-LIM1* was up-regulated in OW, and *Pt-LIM2* was down-regulated. The four MYB TF genes (*Pt-MYB090*, *Pt-MYB156*, *Pt-MYB167* and *Pt-MYB221*) were all up-regulated in OW compared with TW (OW > TW). All the genes encoding TFs that belong to *VNI*, *ASL*, and *KNAT* family members showed the same expression pattern (NW > TW > OW).

### Differential expression of genes involved in hormone responses

In total, 40 genes related to plant hormone signal transduction showed different transcript levels in TW, OW, and NW (Additional file 6), including genes related to auxin, abscisic acid (ABA), ethylene, cytokinin, gibberellin, brassinosteroids, jasmonic acid (JA), and salicylic acid. Of these, eight auxin-related genes were up-regulated in NW compared with TW or OW, and two were down-regulated, *Pt-SAUR1* encoding a SAUR family protein and POPTR_0005s25800 encoding an auxin response factor (Additional file 6). The ABA-related genes encoding serine/threonine-protein kinase (SRK2), protein phosphatase 2C (PP2C), ABA responsive element binding factor, and ABA receptor PYR/PYL family member were all down-regulated in NW compared with TW or OW (Additional file 6). Similar to ABA-related genes, ethylene- and JA-related genes encoding ethylene-insensitive protein, ethylene receptor, MYC2, JA-amino synthetase, and a jasmonate ZIM domain-containing protein were all down-regulated in NW compared with TW or OW (Additional file 6). Most plant hormone signal transduction genes were differentially expressed among TW, NW, and OW (Additional file 6), implying that genes related to hormone signal transduction are involved in the development of reaction wood. Also, three genes involved in hormone biosynthesis, such as *Pt-ACO1.3* related to ethylene, cytokinin oxidase (POPTR_0001s05830) related to cytokinin, and gibberellin 2-oxidase (*Pt-GA2.7*) related to gibberellin, were all down-regulated in TW and NW compared with OW (Additional file 7). Similarly, *Pt-PAP2.3* encoding an auxin-responsive protein and POPTR_0018s06080 encoding an ethylene-responsive transcriptional coactivator were repressed in TW compared with OW (Additional file 7).

### Differential expression of genes involved in lignin, flavonoid, and cellulose biosynthesis

The genes with different transcript levels identified in our study included 33 genes involved in phenylpropanoid biosynthesis (Additional file 6). Of these, 20 genes encoded 11 enzymes, including phenylalanine ammonia-lyase (PAL), cinnamate 4-hydroxylase (C4H), quinate o-hydroxycinnamoyltransferase (HCT), Caffeoyl-CoA O-methyltransferase (CCoAMT), Cinnamoyl CoA reductase (CCR), Coumaroyl 3-hydroxylase (C3H), 4CL, PO, coniferyl aldehyde 5-hydroxylase (CAld5H), cinnamyl alcohol dehydrogenase (CAD), and laccase (LAC) detected (Table 3). The majority of these genes were up-regulated in OW, except *Pt-HCT6, Pt-CAld5H1*, *Pt-CAld5H2, Pt-PO1*, and *Pt-CAD1*, which were up-regulated in TW. Also, five genes encoding proteins involved in monolignol polymerization and modification, such as LAC, were all up-regulated in OW compared with TW (Additional file 6). We also detected 21 differentially transcribed genes involved in the flavonoid biosynthesis pathway (Additional file 6). Most of them were down-regulated in NW compared with OW, such as the gene encoding chalcone synthase (OW > NW > TW), indicating that the majority of genes involved in flavonoid biosynthesis were expressed at the highest levels in OW, then NW, and then TW (OW > NW > TW) (Additional file 6).

**Table 3:** **The differentially-expressed transcripts involved in lignin biosynthesis**

**Gene id**	**Enzyme family**	**Gene name**	**log**_**2**_ **FC(NW/OW)**	**log**_**2**_ **FC(NW/TW)**	**Phytozome ATG**
POPTR_0001s07400	4CL	*Pt-4CL3*	4.27	4.63	AT3G21240.1
POPTR_0003s18720		*Pt-4CL5*	6.52	7.03	AT3G21240.1
POPTR_0006s03180	C3H	*Pt-C3H3*	3.43	3.83	AT2G40890.1
POPTR_0013s15380	C4H	*Pt-C4H1*	3.20	4.47	AT2G30490.1
POPTR_0019s15110		*Pt-C4H2*	1.62	2.40	AT2G30490.1
POPTR_0009s09870	CAD	*Pt-CAD1*	2.08	1.68	AT3G19450.1
POPTR_0005s11950	CAld5H	*Pt-CAld5H1*	6.00	4.64	AT4G36220.1
POPTR_0007s13720		*Pt-CAld5H2*	5.34	4.22	AT4G36220.1
POPTR_0009s10270	CCoAOMT	*Pt-CCoAOMT1*	2.57	3.25	AT4G34050.1
POPTR_0001s31220		*Pt-CCoAOMT2*	2.62	3.87	AT4G34050.1
POPTR_0003s17980	CCR	*Pt-CC-2*	1.38	1.53	AT1G15950.1
POPTR_0012s00670	COMT	*Pt-COMT2*	4.04	3.94	AT5G54160.1
POPTR_0003s18210	HCT	*Pt-HCT1*	2.90	3.75	AT5G48930.1
POPTR_0001s03440		*Pt-HCT6*	5.48	5.14	AT5G48930.1
POPTR_0002s11880	TFs	*Pt-LIM1*	3.84	4.23	AT1G10200.1
POPTR_0014s01590		*Pt-LIM2*	1.16	1.08	AT1G10200.1
POPTR_0015s05130		*Pt-MYB090*	2.92	2.63	AT1G17950.1
POPTR_0009s13640		*Pt-MYB156*	3.47	3.77	AT4G38620.1
POPTR_0012s03650		*Pt-MYB167*	1.90	2.09	AT1G17950.1
POPTR_0004s18020		*Pt-MYB221*	2.91	3.86	AT4G38620.1
POPTR_0006s12870	PAL	*Pt-PAL1*	2.15	5.53	AT2G37040.1
POPTR_0008s03810		*Pt-PAL2*	3.87	5.63	AT2G37040.1
POPTR_0016s09230		*Pt-PAL3*	1.15	4.35	AT2G37040.1
POPTR_0010s23100		*Pt-PAL4*	3.86	4.52	AT2G37040.1
POPTR_0004s01510	Peroxidase	*Pt-PO1*	3.75	2.25	AT4G21960.1
POPTR_0006s13190		*Pt-PO2*	2.44	3.13	AT2G37130.1

Among the differentially expressed genes, 43 genes related to cell wall metabolism were identified, including *cellulose synthase* (*CesA*) (Additional file 6). The majority of these genes were up-regulated in NW compared with TW or OW (Additional file 6). Of these, the cell wall structural protein gene POPTR_0008s01310 encoding a fasciclin-like domain-containing protein, was down-regulated over 8-fold in TW and OW. Among the genes related to the cell wall, we also found two differentially expressed genes encoding sucrose synthase (SUS), *Pt*-*SUS1* (NW > OW > TW) and *Pt*-*SUS2* (OW > TW > NW), and their transcript levels indicated that different *SUS* genes have distinct expression patterns in NW and TW. In total, 11 and 2 genes encoding *CesA* and *COBRA* were found to be differently transcribed, such as *Pt-CESA2.1*, which was up-regulated over 26- and 68-fold in NW compared with TW and OW, respectively (Additional file 6). Also, transcripts of *Pt-CESA2.6*, encoding a protein similar to CesA7A-like, increased over 4-fold in NW compared with TW and OW. Although the transcripts of most of these *CesA* genes were more abundant in NW than TW or OW, the *Pt-MANS.1* and *Pt*-*MANS.2* transcripts were most abundant in TW (TW > NW > OW). POPTR_0014s01060 encoding a protein similar to an *Arabidopsis thaliana* rhamnogalacturonate lyase family protein was up-regulated 1,325 fold in NW compared with OW and did not express in TW, or had so little message that it was undetectable (Additional file 6). One of the two genes encoding COBRA proteins, which are involved in cell wall expansion and/or cellulose deposition, was up-regulated in TW, and the other was down-regulated compared with OW. In addition, 14 genes encoding proteins related to transporters were identified, including a gene encoding a sucrose transporter up-regulated in TW, and two aquaporin genes, *Pt-TIP2.4* and *Pt-PIP2.3*. In particular, nine genes encoding proteins related to cell expansion (expansin-like and expansin-related protein) and cell wall modification (xyloglucan endotransglycosylases, XTHs), S-adenosylmethionine (SAM) were also differentially expressed, including *Pt-EXLB1.1, XTH*, and *Pt-SAM1.2* (Additional file 7).

### Co-expression networks underlying the regulation of lignin and cellulose biosynthesis

**Figure 5:**
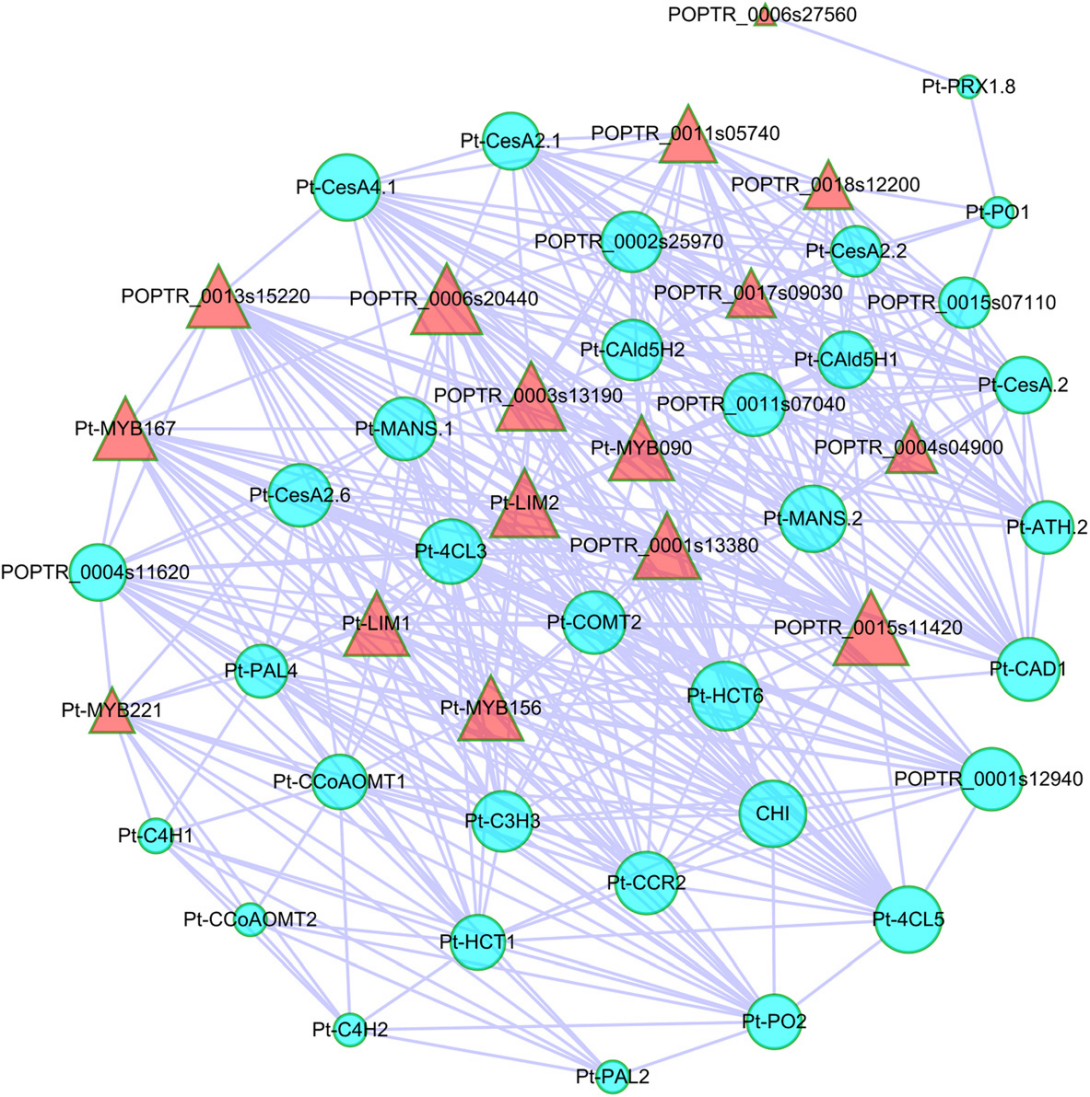
**The co-expression network of significantly differentially expressed genes involved in cellulose and lignin biosynthesis and TFs regulation.** The genes involved in cellulose and lignin biosynthesis are represented by circles and TFs are represented by triangles.

**Figure 6:**
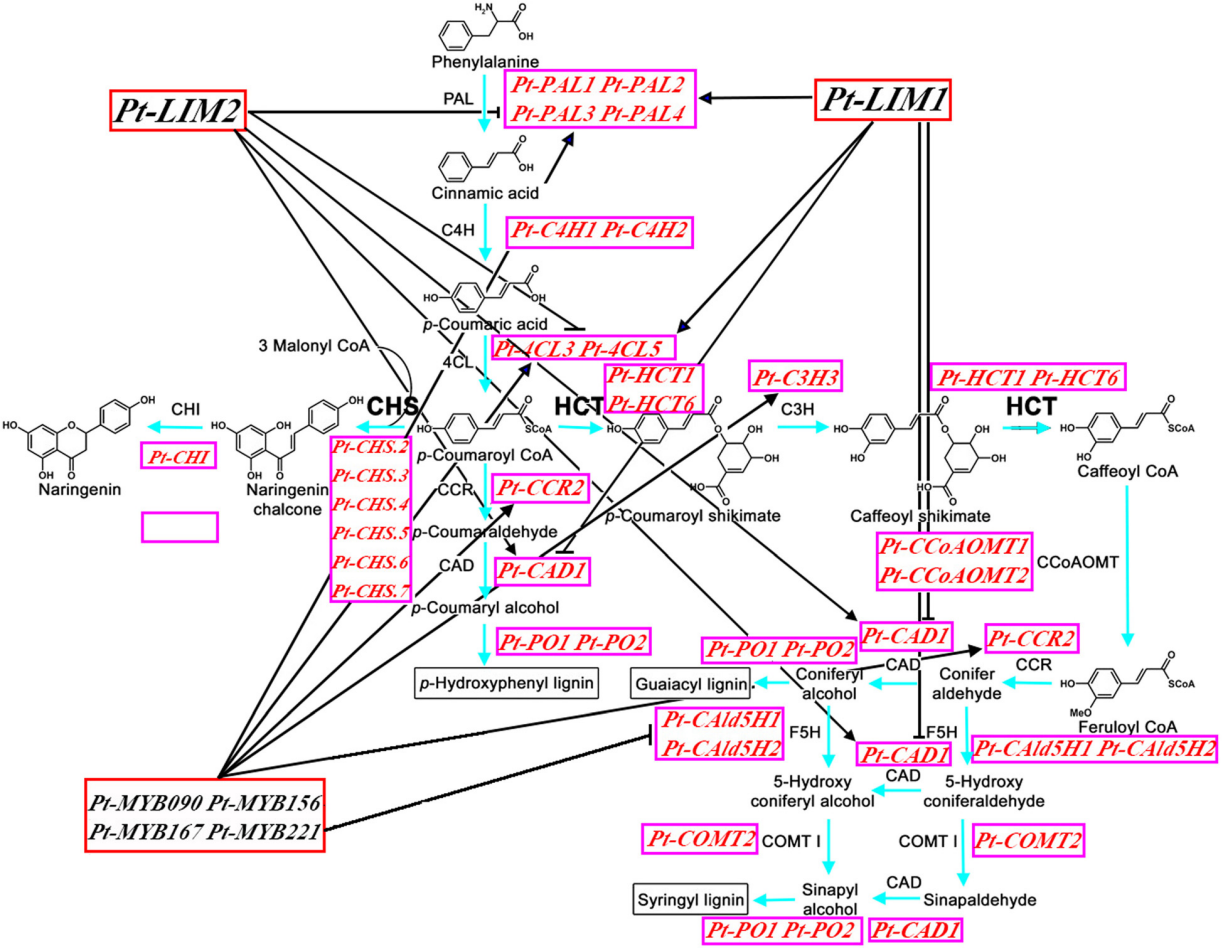
**The potential transcriptomic network regulating lignin biosynthesis.**

**Figure 7:**
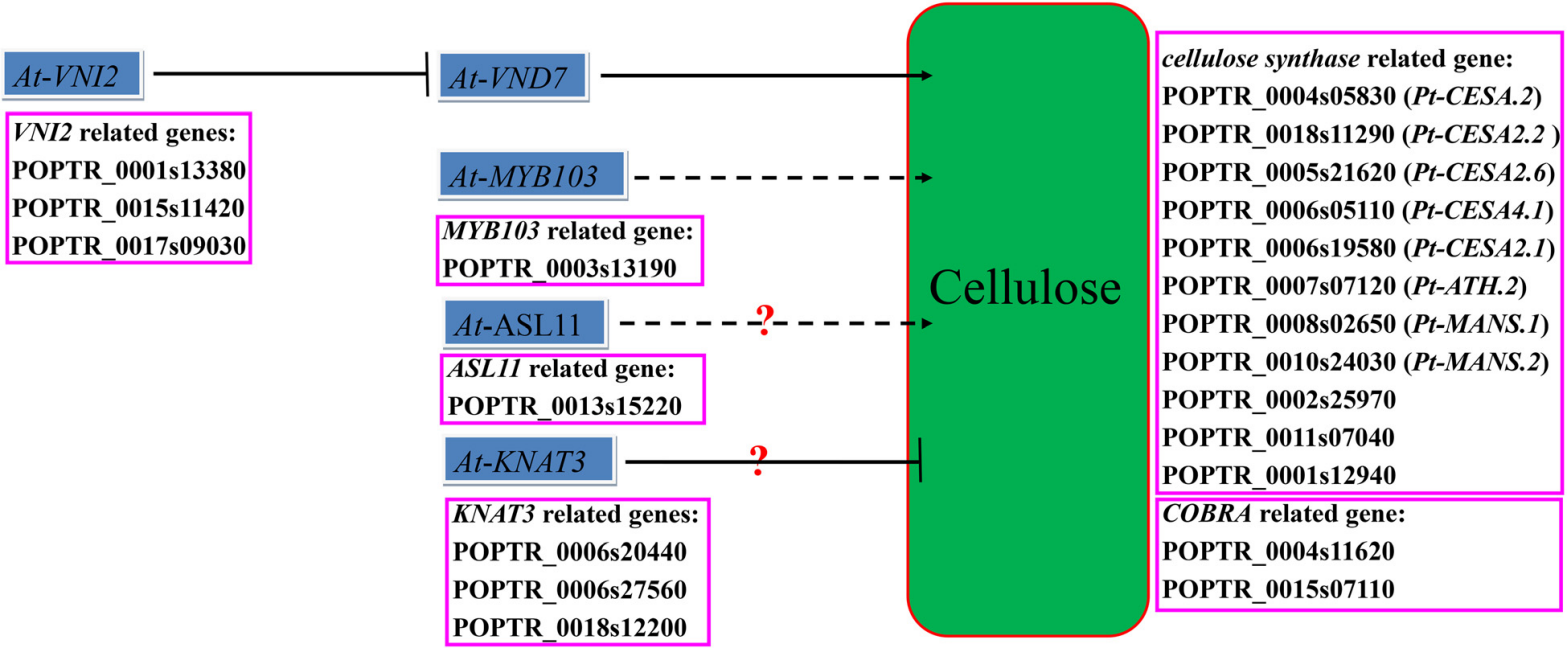
**The potential transcriptomic regulating cellulose biosynthesis.**

To explore the potential correlations of expression of genes involved in cellulose and lignin biosynthesis and regulation, we used our RNA-seq data to construct a global co-expression network (Figure 5) using WGCNA. Our data showed, for example, that TFs such as *Pt-LIM1* and *Pt-LIM2* co-expressed with genes involved in lignin biosynthesis. According to the co-expression of these genes, the differentially expressed genes in NW, TW, and OW, and the TF regulators identified here and in other studies [24], we constructed two transcriptomic networks for the regulation of lignin and cellulose biosynthesis (Figures 6 and 7). The lignin biosynthesis network has 20 genes, including all the genes in the lignin biosynthesis pathway and six TF genes in the LIM and MYB families. The expression patterns of *Pt-MYB090*, *Pt-MYB156*, *Pt-MYB167*, and *Pt-MYB221* (OW > TW) were similar to *Pt-PAL1*, *Pt-PAL2*, *Pt-PAL3*, *Pt-PAL4*, *Pt-4CL3*, *Pt-4CL5*, *Pt-CCR2*, and *Pt-C3H*3 (OW > TW), indicating a co-expression relationship (Figure 5), and a possible direct, positive regulatory relationship (Figure 6). Also, MYB TFs have been reported to regulate *PAL*, *CL*, *CCR*, and *C3H3* [25], but expression of four MYB TF genes was opposite to that of *Pt-CAld5H1* and *Pt-CAld5H2* (TW > OW), suggesting a negative regulatory relationship (Figure 6). For the LIM family, *Pt-LIM1* was up-regulated, and *Pt-LIM2* was down-regulated in OW compared with TW. According to previous studies [26,27], LIM may interact with *PAL*, *CL*, *HCT*, and *CAD*. Considering that *Pt-PAL1*, *Pt-PAL2*, *Pt-PAL3*, *Pt-PAL4*, *Pt-4CL3*, *Pt-4CL5*, and *Pt-HCT1* were up-regulated in OW compared with TW, these genes might have a positive regulatory relationship with *Pt-LIM1* and a negative relationship with *Pt-LIM2* (Figures 5 and 6). However, *Pt-HCT6* and *Pt-CAD1* were down-regulated in OW, similar to *Pt-LIM2*, suggesting they may co-express with *Pt-LIM2* (Figures 5 and 6).

The cellulose biosynthesis network has 13 cellulose synthesis genes, including *CesA* and *COBRA* related genes, and ten TF genes orthologous to *Arabidopsis VNI2* (TW > OW), *MYB103* (TW > OW), *ASL11* (TW > OW), and *KNAT3* (OW > TW) (Figure 7). Since most of the cellulose synthesis genes were up-regulated in TW compared with OW (except POPTR_0002s25970, POPTR_0001s12940, POPTR_0004s11620, and *Pt-CESA4.1*), *KNAT3* may have a negative role in regulating cellulose biosynthesis (Figures 5 and 7). *VNI2*, *MYB103*, and *ASL11* were likely to be co-expressed with the up-regulated cellulose synthesis genes, indicating they might have a positive role in cellulose biosynthesis in TW (Figures 5 and 7).

### qRT-PCR validation of RNA-seq data

**Figure 8:**
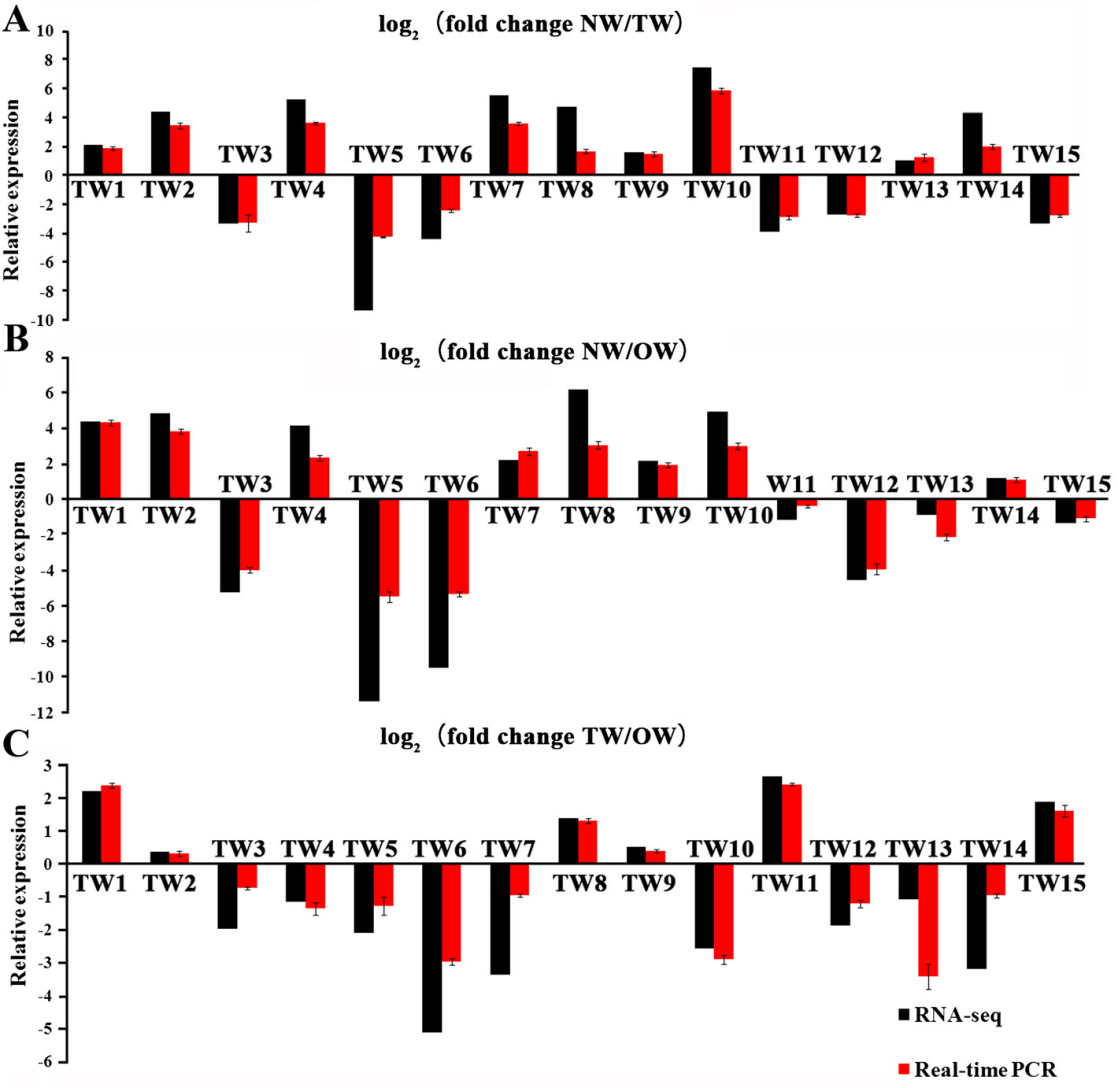
**Comparison of the changes in expression of selected genes detected by RNA-seq and qRT-PCR. A**: Comparison of the changes in expression of selected genes detected by RNA-seq and qRT-PCR among NW and TW. **B**: Comparison of the changes in expression of selected genes detected by RNA-seq and qRT-PCR among NW and OW. **C**: Comparison of the changes in expression of selected genes detected by RNA-seq and qRT-PCR among TW and OW. The values represent log_2_ (fold change) among TW, OW, and NW. Error bars indicate standard deviations.

To verify a subset of the RNA-seq data by an additional independent means, qRT-PCR analyses were conducted. Transcript levels of the 15 genes selected, including two related to cellulose biosynthesis (*Pt-CESA2.1* and *Pt-ATH.2*), and four related to lignin biosynthesis (*Pt-GLAC90.1*, *Pt-PRX1.8*, *Pt-PAL1.2* and *Pt-PAL1.3*), were measured in NW, OW, and TW by using real-time qRT-PCR. The results showed similar expression patterns between RNA-seq and qRT-PCR, thus validating the RNA-seq data (Figure 8).

## Discussion

### Extensive transcriptome remodeling underlies the drastic differences between NW and branch wood

Earlier studies showed that reaction wood, abnormal wood induced by mechanical bending stress or gravity and other natural environmental factors, has many differences with NW [2,13]. Many studies have also uncovered the differences in expression of genes between CW or TW and NW [13,28,29]. These studies revealed important discoveries but also had limitations, in particular because they only considered the differences between NW and TW or between TW and OW [13,28,29]. In this paper, we identified genes differentially transcribed among NW, TW and OW and annotated their various functions in cell division, cell expansion, primary wall synthesis, secondary wall deposition, hormone signaling, transcription, and environmental stresses. Previous studies found drastic differences between NW and TW or TW and OW of *Populus*, indicating that gravity affects cell division, secondary wall deposition, cellulose microfibril orientation, and overall wood properties [2,30,31]. The differences between the TW and OW transcriptomes observed in our study were mostly in agreement with previous data derived from bent trunks of *Populus* [5]. Importantly, we identified 2,658 and 2,417 differentially expressed genes in OW and TW, respectively, in comparison to NW. That shows that more genes were up- or down-regulated in OW than in TW. Thus, these findings support the idea that OW experienced more transcriptome remodeling than TW, resulting in greater phenotypic variation between OW and wood formed in normal conditions (NW), compared to the variation between TW and NW. Our study also detected many metabolic and biosynthetic processes among the significantly enriched GO terms for up-regulated genes in NW compared with TW or OW, suggesting that these processes were mainly repressed in TW or OW. Moreover, all of these results may indicate that the normal biological processes of TW and OW were dramatically affected, compared with NW. Interestingly, the GO terms analysis of differentially expressed genes indicates that the down-regulated genes in NW *vs* OW and NW *vs* TW function in similar processes (Additional file 5). In other words, the genes up-regulated in TW or OW compared with NW are more likely to have the same functions.

Another interesting discovery in our study was that the “molecular function” terms “transcription regulator activity” and “transcription factor activity” were identified among the down-regulated genes in NW compared with TW or OW. Also, more than half of the differentially expressed TF genes decreased over 4-fold in NW, indicating that TF abundances differed dramatically in these tissues; these TFs may have important roles in reaction wood (Additional file 6). Furthermore, the term “transcription repressor activity” was detected among the genes down-regulated in NW compared with TW, suggesting that repressors were up-regulated in TW and may repress the expression of their target genes [32]. Previous work found many differentially regulated TFs in developing TW in *P. tremula* (L.) × *tremuloides* (Michx.) TW-forming tissues induced by bending field-grown trees compared to normal wood [5]. Since TFs can recruit coactivator or corepressor proteins to a transcription factor DNA complex [33], the TFs up-regulated in reaction wood may play positive or negative roles in reaction wood formation. In addition, our results indicated that several classes of genes encoding HSPs were mostly up-regulated in TW. HSPs maintain protein homeostasis inside cells and promote the proper folding, stability, and degradation of polypeptides [34]. Moreover, previous studies have suggested a role for HSPs in early xylem development in wood in *P. tomentosa*, thereby emphasizing the importance of HSP genes as candidate genes for the genetic improvement of wood quality [4]. Extended cell wall thickening and a delay of programmed cell death were observed with the proteins, including HSPs [4]. Here, the genes encode HSPs were up-regulated in TW, which may reflect stronger cell wall thickening and delays of programmed cell death in TW, compared with OW.

### Ehylene and gibberellin have potential regulatory roles in reaction wood formation

The plant hormones have important roles in wood development and have been studied for decades [10,35]. A previous study used microarrays to examine the differentially transcribed genes in CW and OW in radiate pine branches and showed that many genes related to hormones and calcium signaling, as well as various environmental stresses, were exclusively up-regulated in CW [13]. For *Populus*, the up-regulation of *1-Aminocyclopropane-1-carboxylate oxidase* (*ACO*) in TW compared with NW was detected in hybrid aspen [36]. In our study, *Pt-ACO7*, encoding a putative ACO, was down-regulated in NW compared with either TW or OW and was down-regulated in TW compared with OW (Additional file 7). It could be proposed that the abundance of ACO is highest in OW, then TW and NW (OW > TW > NW), which may indicate that ethylene participates in TW formation. Ethylene interacts synergistically with gibberellins and cytokinin in TW formation in *Leucaena leucocephala* [36] and the formation of TW in *A. mangium* seedlings requires gibberellin, especially gibberellin 2-oxidase [10]. Consistent with this, *Pt-GA2.7* encoding a protein similar to gibberellin 2-oxidase was up-regulated to its highest levels in OW (Additional file 7). In summary, we have identified many genes related to hormone signaling and biosynthesis, as well as environmental stresses, as differentially transcribed in NW, TW, and OW. This provides valuable clues to improve our understanding of reaction wood formation in response to gravity stimulus. Overall, our results that show ethylene and gibberellin have potential regulatory roles in reaction wood formation.

### Transcriptomic networks underlying the regulation of lignin and cellulose biosynthesis in NW, TW, and OW

Previous studies revealed that TW has more cellulose and less lignin than OW or NW [30,31]; in accordance, genes in the phenylpropanoid pathway for biosynthesis of lignin monomers and flavonoids were greatly down-regulated [5,29]. In our study, transcript levels of 33 genes encoding 11 enzymes of the lignin biosynthetic pathway, including CCoAOMT, PAL, C4H, CAM, CCR, C3H, 4CL, PO, CAld5H, CAD, and LAC, were lower in TW compared with OW (Table 3). This is consistent with previous work showing that poplar TW has lower expression of *PAL*, *4CL*, and *CAD* [5]. The genes encoding LACs, which are involved in the polymerization of monolignols to produce lignin macromolecules, were also significantly down-regulated in TW [29]. Similar results were found in our study, as five genes encoding proteins like LAC were all down-regulated in TW compared with NW; this also agrees with a report showing that expression of these genes decreased during TW formation in *Populus* [5]. Our results confirm that genes in the phenylpropanoid pathway affecting biosynthesis of both lignin monomers and flavonoid were greatly down-regulated in TW compared with OW. This could explain the lower lignin content in TW compared with OW.

In recent years, many studies have examined the genes involved in lignin biosynthesis [37], and the regulatory network underlying lignin biosynthesis is gradually being revealed to include TFs, specific expression of gene family members, and control by pathway intermediates [38]. Previous studies reported that TFs including LIM [26,27] and MYB [25] family members could regulate the genes involved in lignin biosynthesis and affect the lignin content or lignin monomer ratio. Further study revealed that plant LIM domain proteins may act as transcriptional activators of lignin biosynthesis, activating the expression of *PAL*, *4CL*, and *CAD* and/or function as actin binding and bundling proteins [26]. Also, the expression pattern of the twelve duplicated poplar *Ptr-LIM* (*P. tremula* × *P. alba*) genes investigated by qRT-PCR showed that poplar *Ptr-XLIM1a*, *Ptr-XLIM1b*, and *Ptr-WLIM1b* were preferentially expressed in the secondary xylem, including TW and OW, suggesting a specific function in wood formation and mechanical stress response [39]. According to the differential expression of two LIM TFs observed in our study, *Pt-LIM1* and *Pt-LIM2*, it is tempting to speculate that they may have roles in wood formation and may also function in the response to mechanical stress (Figure 6). For the MYB family, a recent study reported that when *Pt-MYB216* (*P. tomentosa*) was overexpressed, *PAL4*, *4CL5*, *C3H3*, and *CCR2* were up-regulated and *CAld5H* was down-regulated, but no obvious changes occurred in transcript levels of *CCoAOMT1*, *COMT2*, and *CAD1*, demonstrating that *Pt-MYB216* is a transcriptional activator of lignin biosynthesis during secondary wall formation [40]. In our study, four MYB TFs (*Pt-MYB090*, *Pt-MYB156*, *Pt-MYB167*, and *Pt-MYB221*), which probably regulate lignin biosynthesis, were all up-regulated in OW compared with TW. However, few prior studies have constructed a possible regulatory network to examine lignin biosynthesis. Here, our study provided a transcriptomic regulatory network including TFs and enzymes of the lignin biosynthetic pathway (Figure 6) via a co-expression network among TW, OW, and NW; this network may enable the analysis of the regulation of lignin biosynthesis. The co-expression analysis found that *Pt-MYB090*, *Pt-MYB156*, *Pt-MYB167*, and *Pt-MYB221* may co-express with *Pt-PAL1*, *Pt-PAL2*, *Pt-PAL3*, *Pt-PAL4*, *Pt-4CL3*, *Pt-4CL5*, *Pt-CCR2*, and *Pt-C3H*3, and have a negative expression relationship with *Pt-CAld5H1* and *Pt-CAld5H2*. Also, we found that *Pt-LIM1* co-expressed with *Pt-PAL1*, *Pt-PAL2*, *Pt-PAL3*, *Pt-PAL4*, *Pt-4CL3*, *Pt-4CL5*, and *Pt-HCT1* and *Pt-LIM2* co-expressed with *Pt-HCT6* and *Pt-CAD1*. Together with previous studies [25-27], these findings indicate that the transcriptomic regulatory networks (Figure 6) provided in our study reflect the lignin biosynthetic pathway regulated by TFs.

The gradual exploration of cellulose biosynthesis has identified more and more genes affected cellulose biosynthesis, including *CesA*s [41], *KORRIGAN* [42], *SUS* [43], *COBRA* [44], and various TFs [45,46]. Previous studies showed the factors (hormones, light, mechanical stimuli, nutrition, and interactions with the cytoskeleton) that influence cellulose deposition by affecting the levels of substrate, and the abundance of cellulose synthase complexes (CSCs). Different *CesA* have different functions and different expression patterns [41,47], especially in the primary and secondary cell wall. In the tension stress-responsive genes examined in aspen, *CesA* genes, namely *PtrCesA1*, *PtrCesA2*, and *PtrCesA3*, which are closely associated with secondary cell wall development in the xylem cells, also exhibited similar tension stress-responsive behavior [42]. In another study of TW genes in *Populus* by microarray and metabolite analysis, the *PttCesA8-2* and *PttCesA3-2* transcripts were found to be up-regulated in TW compared with NW [5]. Interestingly, the “cellulose metabolic process” and “cellulose biosynthetic process” terms were also found in to be enriched in up-regulated genes in NW compared with TW or OW in our study. However, for the “molecular function” terms, “cellulose synthase (UDP-forming) activity” and “cellulose synthase activity” were only found to be enriched among the up-regulated genes in NW *vs* OW, showing that the genes related to *CesA* were down-regulated in OW. Importantly, nine *CesA* related genes were up-regulated in NW, and two genes (*Pt-MANS.1* and *Pt-MANS.2*) were up-regulated in TW in our study. Together with previous studies [5,42], this supports the idea that different *CesA*s might be present in the biosynthetic complexes of TW and NW. Similar to *CesA*s, *SUS*s have an important role in cellulose synthesis, and overexpression of a cotton *SUS* gene in poplar resulted in increased cellulose synthesis, supporting the idea of a direct connection between sucrose supply, sucrose breakdown, and cellulose production through *SUS* [48]. Interestingly, different *SUS*s have different effects, since overexpression of a bean *SUS* sequence in poplar did not increase the amount of cellulose [49], and another study in pea indicated that different *SUS*s are associated with different metabolic fates of sucrose [50]. Consequently, the two differentially expressed *SUS* genes, *Pt*-*SUS1* (NW > OW > TW) and *Pt*-*SUS2* (OW > TW > NW), identified in our study have opposite expression patterns, which may indicate that different *SUS*s have different functions in cellulose biosynthesis. COBRAs are involved in cell wall expansion and cellulose deposition by affecting cellulose microfibril orientation during plant morphogenesis [51]. *AtCOBL4* is required for cellulose biosynthesis in the secondary wall and was identified by the similarity of its expression to *Arabidopsis AtCesA*s implicated in secondary cell wall synthesis [52,53]. However, little is known about *COBRA*s, and the differentially expressed *COBRA* related genes POPTR_0004s11620 and POPTR_0015s07110 may be useful in determining the function of *COBRA*s. In summary, our study identified a series of differentially expressed genes involved in cellulose biosynthesis and suggested more complicated functions of different members.

Taking advantage of genome-wide changes, transcriptome profiling of wood formation has identified a number of TFs that are preferentially expressed in developing wood and may regulate gene expression [25,54,55]. Among these, a set of MYB TFs that are functional orthologs of *Arabidopsis* TFs has been shown to be involved in the regulation of secondary wall biosynthesis during wood formation in *Populus* [25,54,55]. In detail, *PtrMYB3* and *PtrMYB20*, functional orthologs of *Arabidopsis MYB46* and *MYB83*, can activate the biosynthetic pathways of cellulose, xylan, and lignin, suggesting that they regulate the biosynthesis of all three major wood components in poplar [55]. Our study found a positive relationship between *Pt-MYB103* expression and expression of most of the cellulose synthesis genes, suggesting a positive role for *Pt-MYB103*. For NAC domain TFs, the positive master regulator of secondary cell wall biosynthesis in *Arabidopsis* including the functionally redundant VASCULAR-RELATED NAC-DOMAIN7 (VND7) and VND6 TFs function in secondary cell wall formation in protoxylem and metaxylem [56,57]. Previous work revealed that these NAC domain TFs can directly activate the expression of secondary wall-specific biosynthetic genes and activate the expression of several downstream transcription factor genes that also directly regulate genes involved in the biosynthesis of secondary wall components [55,58]. Our study found that the three TF orthologs of *Arabidopsis VNI2* co-express with most of the cellulose synthesis machinery genes. However, few studies have explored the role of NAC domain and KNAT TFs in *Populus* wood formation [59]. Considered negative regulators, the Class II KNOX gene *KNAT7* negatively regulates secondary wall formation in *Arabidopsis* and is functionally conserved in *Populus* [45,60]. The *KNAT3* related genes (Figure 7) were co-expressed with the down-regulated cellulose synthesis machinery genes, which supports the idea that they have a negative role in TW cellulose biosynthesis. For trees, few advances have been achieved in understanding the regulation of cellulose biosynthesis; this requires more studies to uncover the molecular mechanism [24]. The possible regulatory network of cellulose biosynthesis genes and TFs described here may help us to understand the complicated regulation of cellulose biosynthesis (Figure 7). Understanding the complex process of wood formation will be important for optimizing the use of wood as a renewable energy source. Although significant progress has been made in understanding the cellulose and lignin synthesis machinery [24], many fundamental aspects remain to be addressed. Wood formation or secondary cell wall formation is an exciting area of study with many challenges and opportunities. Uncovering the transcriptional regulators controlling wood biosynthesis will provide novel tools to alter the biosynthetic pathways of wood components based on our needs.

In addition, apart from the genes directly involved in lignin and cellulose biosynthesis, many genes related to cell wall also have important roles in reaction wood formation, particularly several *expansin*, *XTH*, and *SAM* genes, which were differentially transcribed in our study. The quantity of wood formation is largely related to cell division and expansion during primary cell wall development. Expansins are involved in cell expansion in all tissues of the plant and have been isolated from the secondary xylem [61]. Differential expression of *expansin*s in TW has been reported [5]. In our study, the genes encoding expansin-like and expansin-related protein were differentially expressed between TW and OW, for example, *Pt-EXLB1.1* was up-regulated in OW (Additional file 7). An earlier study confirmed that *XTH*s function during the formation of secondary cell walls of vascular tissues and are believed to be important regulators of primary wall expansion [13]. Our study showed the up-regulation of *XTH* in TW compared with OW (Additional file 7). Differential transcription of these genes between TW and OW could provide a molecular explanation for the similar tracheid diameters (either radial or tangential directions) in these two types of wood. A previous study found the transcripts of *SAM* in the developing xylem and showed that SAM is important in methylation reactions during biosynthesis of coniferyl and sinapyl alcohols [62]. Differences in the availability of SAM may affect wood quality by altering lignin content and composition [63]. Recent studies have revealed that *SAM*s were up-regulated in CW [13,29] in conifer. However, we have little information on *SAM*s in *Populus*. In our study, *Pt-SAM1.2* mRNA levels increased in the OW, which may point to a role for *SAM*s in reaction wood formation. All together, genes involved in primary wall modification, wood growth, and tracheid dimensions were differentially expressed in branch wood of *Populus*, providing more clues to the molecular mechanisms affecting wood formation.

## Conclusions

This genome-wide transcriptome profiling of branch (TW and OW) and normal stem wood (NW) from *P. tomentosa* provides more accurate insights into the molecular basis of TW formation in response to gravity stimulus by identifying differentially expressed genes using RNA-Seq. In total, 3,058 significantly differentially expressed genes were identified, including genes involved in secondary cell wall structure and wall modification and composition, such as cellulose and lignin biosynthetic genes. Two transcriptomic networks that underlie lignin and cellulose biosynthesis regulation were constructed, including TFs, based on the co-expression network, which may provide clues for understanding the regulation of cellulose and lignin biosynthesis. However, many key differentially expressed genes identified in our study were poorly annotated, which impedes our understanding of the molecular mechanism of wood formation. Importantly, the high-resolution expression patterns presented here improve our understanding of the molecular mechanisms and expand our knowledge of reaction wood formation in *Populus*. These findings may have potential applications for the improvement of wood properties in plants via genetic engineering.

### Availability of supporting data

The Illumina reads have been deposited in the Sequence Read Archive at NCBI (http://www.ncbi.nlm.nih.gov/sra) and they are available under study accession number SRP040531. And the sequences of 44 genes involve in cellulose and lignin biosynthesis regulation pathways were uploaded in NCBI GenBank (http://www.ncbi.nlm.nih.gov/nuccore/) with accession number KP769977-KP770020.

## Additional files

Additional file 1:
**The primers used in this study.**
Additional file 2:
**The information and expression of transcripts in the three libraries.**
Additional file 3:
**Gene expression in the three libraries.**
Additional file 4:
**The differentially expressed genes.**
Additional file 5:
**The significant GO terms of differentially expressed genes.**
Additional file 6:
**The different genes discussed in the paper.**
Additional file 7:
**The important differentially expressed genes between TW and OW.**
