# Supplementary material for: Transcript profiling of Populus tomentosa genes in normal, tension, and opposite wood by RNA-seq
Source: BMC Genomics. 2015 Mar 10;16(1):164. doi: 10.1186/s12864-015-1390-y (PMC4372042; doi:10.1186/s12864-015-1390-y)
Supplement: Additional file 1: — The primers used in this study. [file 12864_2015_1390_MOESM1_ESM.doc]

**Supplementary material 1** The primers used in this study

| **ID** | **NO.** | **Primer sequence (5' - 3')** |
| --- | --- | --- |
| **POPTR_0001s18730** | Twq1 | Forward: 5'-GGATCCCTCGGCACCATAG -3' (58bp, Tm=60°C) |
|  |  | Reverse: 5'-AGATGTTGGCACCCACAATGA -3' |
| **POPTR_0001s29670** | Twq2 | Forward: 5'-CTACCAGCCACCCACAGTTGT -3' (60 bp, Tm=60°C) |
|  |  | Reverse: 5'-CACACAGCCCTCTGGACCTT -3' |
| **POPTR_0001s38760** | Twq3 | Forward: 5'-AAGAGTGTCAGGCACAGGGTTT -3' (72 bp, Tm=56°C) |
|  |  | Reverse: 5'-TGGTCCACCAAAATAAATCATTGA -3' |
| **POPTR_0002s19000** | Twq4 | Forward: 5'-TGTCAAGCCCCTCAAATGG -3' (63 bp, Tm=57°C) |
|  |  | Reverse: 5'-CGCAAAAGGGATAGGATAAATCA -3' |
| **POPTR_0003s08140** | Twq5 | Forward: 5'-GGCCCAAGCAGCAAATGTTA -3' (68 bp, Tm=59°C) |
|  |  | Reverse: 5'- GCTGAACGGTAGAGTCATAAGCAA-3' |
| **POPTR_0005s14190** | Twq6 | Forward: 5'-CTTGACTATGAGGTGCCTTGAAAA -3' (71bp, Tm=56°C) |
|  |  | Reverse: 5'-CGGTAGTGACGACATTTGCAA -3' |
| **POPTR_0006s12870** | Twq7 | Forward: 5'-GTTGGGAACTGGATTGCTCACT -3' (73 bp, Tm=60°C) |
|  |  | Reverse: 5'-GGCTGTAAACACCTTGTCGAACT -3' |
| **POPTR_0006s19580** | Twq8 | Forward: 5'- TTTGCCTTCTGGGTGATTGTC-3' (61 bp, Tm=58°C) |
|  |  | Reverse: 5'-TTTGCCTCCCCATTAGACCTT -3' |
| **POPTR_0007s07120** | Twq9 | Forward: 5'-CGCCTTGTGGGTCATCATC -3' (71 bp, Tm=60°C) |
|  |  | Reverse: 5'-TGGGCATGCGATCTTGTTT -3' |
| **POPTR_0008s07370** | Twq10 | Forward: 5'-GTTGTTCTTCCCTGCATTTGG -3' (87 bp, Tm=56°C) |
|  |  | Reverse: 5'-GATGGCAGCTCTTGTTTCTAAGG -3' |
| **POPTR_0008s15740** | Twq11 | Forward: 5'-CTTGGGTTCCAGTACAGAGTTGAG -3' (66 bp, Tm=58°C) |
|  |  | Reverse: 5'- CCAGCGAAGCAACAAGGAAT-3' |
| **POPTR_0011s00970** | Twq12 | Forward: 5'-TTCTACAACCCAGGCAGTGATG -3' (68 bp, Tm=59°C) |
|  |  | Reverse: 5'-TCCTCTGCTTCCTTCTCTACCAA -3' |
| **POPTR_0013s08110** | Twq13 | Forward: 5'-CCCAACAAGCTGGTACTGCAT -3' (68 bp, Tm=57°C) |
|  |  | Reverse: 5'-AAGTGATGATCCTTGCTTTGCTTT -3' |
| **POPTR_0016s09230** | Twq14 | Forward: 5'-CCTGCAGTGCTACCTATCCATTG -3' (63 bp, Tm=58°C) |
|  |  | Reverse: 5'-CCAACGCATGGTCAACAAAA -3' |
| **POPTR_0018s06080** | Twq15 | Forward: 5'-CGCGGCTGGAGAAGAAGAT -3' (61 bp, Tm=59°C) |
|  |  | Reverse: 5'-TTTGGCTGCTCATTAATCAGCTT -3' |
| ***ACTIN*** |  | Forward: 5'- CTCCATCATGAAATGCGATG -3' |
|  |  | Reverse: 5'- TTGGGGCTAGTGCTGAGATT -3' |
